# Tracked ultrasound registration for intraoperative navigation during pediatric bone tumor resections with soft tissue components: a porcine cadaver study

**DOI:** 10.1007/s11548-023-03021-x

**Published:** 2023-11-04

**Authors:** J. M. van der Zee, M. Fitski, M. A. J. van de Sande, M. A. D. Buser, M. A. J. Hiep, C. E. J. Terwisscha van Scheltinga, C. C. C. Hulsker, C. H. van den Bosch, C. P. van de Ven, L. van der Heijden, G. M. J. Bökkerink, M. H. W. A. Wijnen, F. J. Siepel, A. F. W. van der Steeg

**Affiliations:** 1 Princess Máxima Center for Pediatric Oncology, Utrecht, The Netherlands; 2 Technical Medicine, TechMed Centre, University of Twente, Enschede, The Netherlands; 3 Department of Orthopaedics, Leiden University Medical Center, Leiden, The Netherlands; 4 Department of Surgical Oncology, Netherlands Cancer Institute, Amsterdam, The Netherlands; 5 Robotics and Mechatronics, TechMed Centre, University of Twente, Enschede, The Netherlands

**Keywords:** Osteosarcoma, Ewing sarcoma, Pediatric oncology, Image-guided surgery, Tracked ultrasound, Intraoperative navigation

## Abstract

**Purpose:**

Resection of pediatric osteosarcoma in the extremities with soft tissue involvement presents surgical challenges due to difficult visualization and palpation of the tumor. Therefore, an adequate image-guided surgery (IGS) system is required for more accurate tumor resection. The use of a 3D model in combination with intraoperative tracked ultrasound (iUS) may enhance surgical decision making. This study evaluates the clinical feasibility of iUS as a surgical tool using a porcine cadaver model.

**Methods:**

First, a 3D model of the porcine lower limb was created based on preoperative scans. Second, the bone surface of the tibia was automatically detected with an iUS by a sweep on the skin. The bone surface of the preoperative 3D model was then matched with the bone surface detected by the iUS. Ten artificial targets were used to calculate the target registration error (TRE). Intraoperative performance of iUS IGS was evaluated by six pediatric surgeons and two pediatric oncologic orthopedists. Finally, user experience was assessed with a post-procedural questionnaire.

**Results:**

Eight registration procedures were performed with a mean TRE of 6.78 ± 1.33 mm. The surgeons agreed about the willingness for clinical implementation in their current clinical practice. They mentioned the additional clinical value of iUS in combination with the 3D model for the localization of the soft tissue components of the tumor. The concept of the proposed IGS system is considered feasible by the clinical panel, but the large TRE and degree of automation need to be addressed in further work.

**Conclusion:**

The participating pediatric surgeons and orthopedists were convinced of the clinical value of the interaction between the iUS and the 3D model. Further research is required to improve the surgical accuracy and degree of automation of iUS-based registration systems for the surgical management of pediatric osteosarcoma.

**Supplementary Information:**

The online version contains supplementary material available at 10.1007/s11548-023-03021-x.

## Introduction

Osteosarcoma (OS) is a rare primary malignant bone tumor in children and young adults with an annual incidence of 8–11 cases per million persons at 10–19 years of age [1, 2]. The disease is commonly found in the extremities, specifically the proximal tibia, distal femur and proximal humerus, and has an overall five-year survival rate ranges between 50 and 66% in the Netherlands [3]. Treatment involves neoadjuvant chemotherapy, surgical resection of the tumor and subsequent adjuvant chemotherapy. The best surgical approach is based on local tumor extension, neurovascular structure involvements and the wishes of the patients and their caregivers. Nowadays, limb salvage surgery is possible in ~ 90% of patients with OS in the extremities, while in the remaining cases amputation is still indicated [4]. Although limb salvage surgery improves the patient’s quality of life by maintaining the functionality of the limb, it can pose surgical challenges due to the need for negative resection margins. Incomplete resections increases the risk of local recurrence significantly [5]. Furthermore, soft tissue involvement of the tumor is often difficult to visualize or palpate, which requires extensive preoperative surgical planning.

To overcome these surgical challenges, image-guided surgery (IGS) is used in adult orthopedic surgery [6]. This may improve surgical and oncological outcomes and eventually may reduce operating time. Importantly, IGS can aid in safe oncological margins while maintaining as much healthy tissue as possible, minimizing morbidity [5]. Nevertheless, the implementation of IGS during the localization of soft tissue components is currently limited [7]. Commercialized IGS systems often use pre-incisional registration to match the virtual model with the physical patient. This registration method requires the insertion of Kirschner wires into the bone to attach a rigid reference body for tracking. Secondly, an additional pre-incisional computed tomography (CT) scan is made that interrupts the surgical workflow for approximately 15 min and the pediatric patient is exposed to additional harmful radiation [8]. Alternative registration methods could result in a faster image acquisition and lower radiation exposures for the patient. Also, if surgical staff bumps into the reference bodies and the registration becomes inaccurate during surgery, which is considered as a frequent problem with conventional IGS systems, quick and fast re-registration must be easily accessible.

Intraoperative tracked ultrasound (iUS) combines image acquisition with real-time positional information. The iUS can be used to find rigid anatomical structures which are required for registration. Bone surface on US gives a unique appearance due to a large impedance difference between soft tissue and the hard bone surface. Therefore, automatic segmentation of the bone surface on iUS followed by 3D volume reconstruction can serve as registration features [8–12]. However, the clinical value of the proposed IGS system during OS resection, especially with the involvement of soft tissue components, has not been shown yet. In this study, an iUS-based IGS system was developed and the proof of principle of an iUS-based registration system was subsequently evaluated in a porcine cadaver study.

## Method

### Experimental setup

**Fig. 1:**
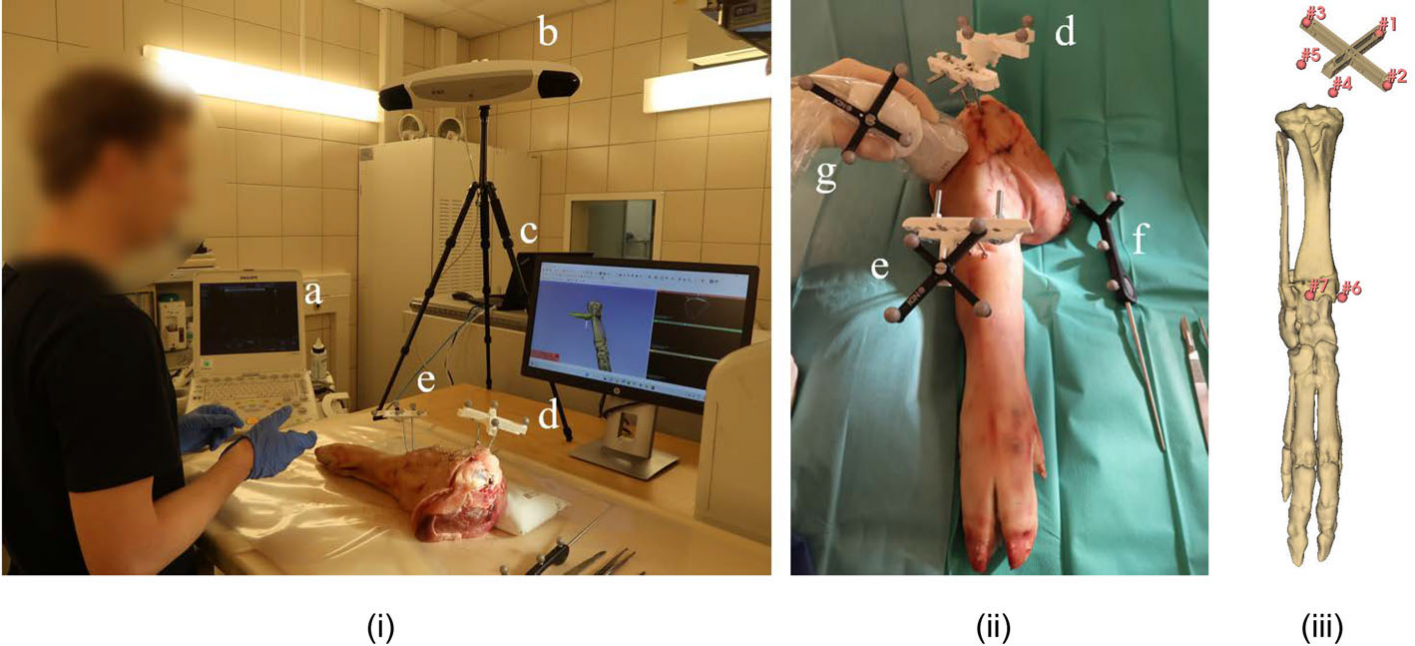
**i** The experimental setup and the **ii** intraoperative situation with (a) an ultrasound machine and frame grabber, (b) optical tracker, (c) 2D interactive screen, (d) ground truth optical reference body, (e) cadaver optical reference body, (f) tracked surgical pointer and (g) tracked ultrasound probe. **iii** Registration points in the 3D model

iUS imaging was performed with a Philips CX50 US (Philips, Best, the Netherlands) machine with a linear probe (Fig. 1a). The US images were streamed with a frame grabber (Epiphan System Inc., Ottawa, Canada) to a computer workstation (Intel® UHD Graphics 16 GB graphical card, 32 GB Ram) (Fig. 1c). Positional data of the US probe, surgical instruments and cadaver were captured by an optical tracking system (Northern Digital Inc., Polaris Vega ST, Waterloo, Ontario, Canada) (Fig. 1b). An optical reference body (ORB) was attached to the US probe with a 3D-printed clip (Fig. 1g). The open-source PLUS toolkit was used to stream both the tracking and imaging data to open-source 3DSlicer and SlicerIGT extension [13]. A calibration procedure between the pixel coordinates and the ORB was performed with a tracked needle calibration method [14]. The tracked surgical pointer (Fig. 1f) was calibrated through a pivot calibration procedure in 3DSlicer.

### Automatic segmentation

A fully automatic bone surface segmentation algorithm was developed using a 2D U-Net network [15]. This network was trained and tested with 1085 B-mode US images with an 80:20 train/test split, using a binary cross-entropy combined Dice loss function and hyper-parameter optimization. A batch size of 4 and an Adam optimizer with a learning rate of 1e^−4^ were considered optimal. The included US images were acquired from the tibia, femur and humerus of four volunteers. Data augmentation of the trainings set was applied by adding randomly a Gaussian blur (sigma = 1.1) and a left–right flip for a quarter of the total set. Labeling of the dataset was done manually by an experienced technical physician.

The network was subsequently validated on an independent dataset that included 942 images acquired from the tibia, femur, ribs, sternum and humerus of two other volunteers. Finally, the network was evaluated based on the performance of the detection of the bone surface by determining the largest component in the prediction of the network. The Dice similarity coefficient (DSC) and the coverage percentage were used to measure the performance.

### Cadaver experiment

The IGS method was validated using eight lower limbs derived from porcine cadavers. These cadavers were CT scanned (Siemens, Erlangen, Germany) with a fixated ORB that served as a gold standard (Fig. 1d). The preoperative 3D model of the bones was derived with threshold segmentation. Ten artificial surgical targets were digitally defined on the bone surface, and one sphere-shaped tumor was placed in the proximal tibia within this 3D model.

### Registration

A gold standard and an iUS rigid registration procedure were performed. The gold-standard registration was done using a point-based registration following an iterative closest point (ICP) method. Digital and physical points were found by the surgical assistant on the preoperative imaging and assigned on the physical model with the tracked surgical pointer. These points were located at the screw heads, Kirschner wires and three pivot points on the 3D-printed frame (Fig. 1c).

**Fig. 2:**
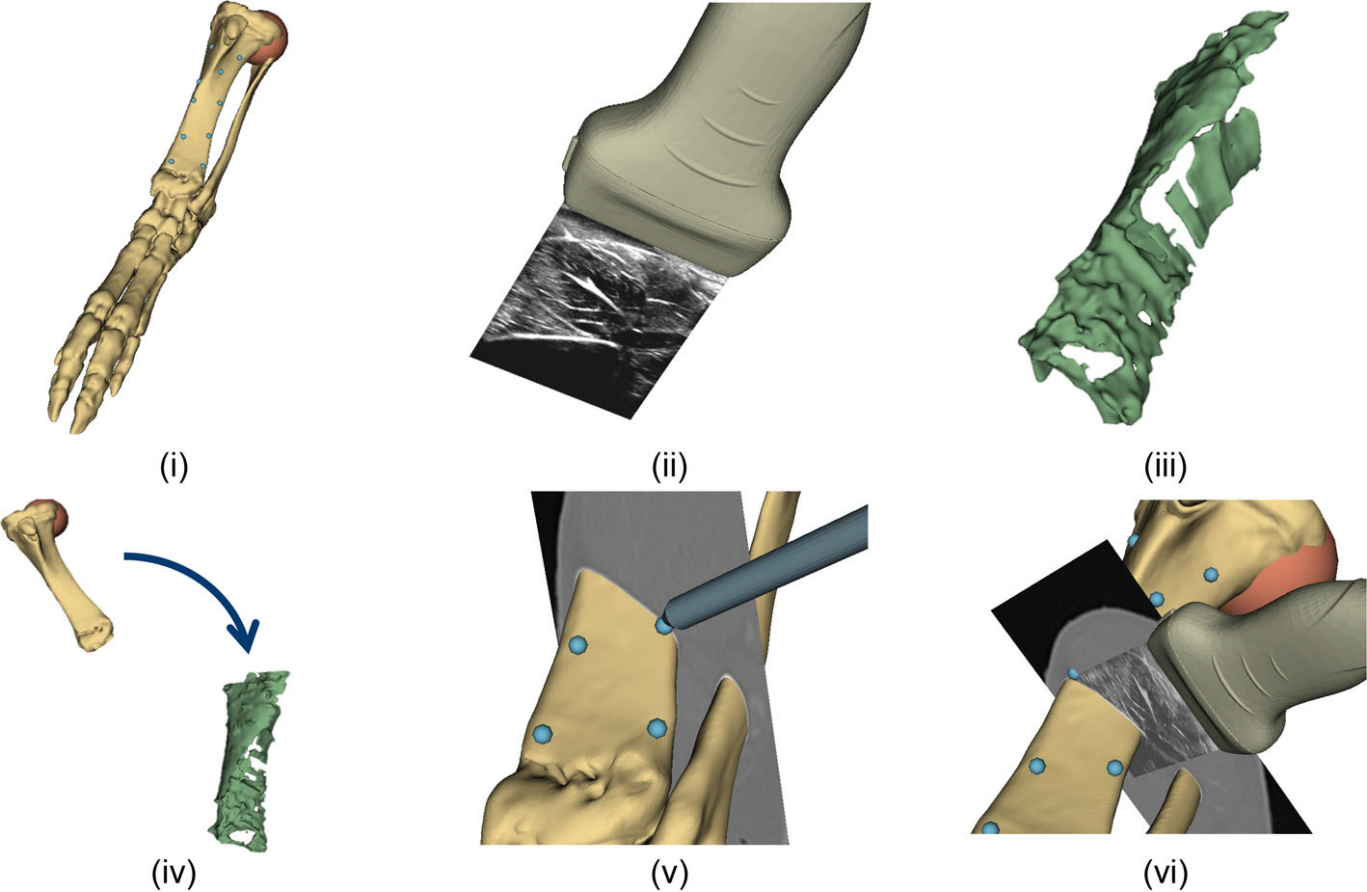
Overview of the registration method used by the iUS-based registration system. **i** The preoperative derived 3D model with the artificial sphere-shaped tumor in the proximal tibia and the artificial surgical targets (blue dots). **ii** iUS acquisition. **iii** The iUS-derived bone surface after automatic segmentation and 3D volume reconstruction. **iv** The model-to-model registration between the virtual planning (CT) with the physical cadaver (US). Intraoperative navigation with the 3D model and tracked surgical pointer **v** and with the iUS **vi** superimposed with CT

The iUS-based registration consisted of six steps (Fig. 2). First, a second ORB (Fig. 1e) was fixated in the proximal tibia. Within this reference frame, a pre-incisional iUS sweep was performed on the skin of the cadaver (Fig. 2ii). Secondly, the bone surface on every slice was detected by the automatic segmentation algorithm. Thirdly, the 3D bone surface was derived after 3D volume reconstruction with volume reconstruction in 3DSlicer (Fig. 2iii) [13]. Protrusions in the volume were removed by manually selecting the biggest volume. The volume was not smoothed during post-processing. Finally, the preoperative 3D model based on the CT was registered by the surgical assistant to this intraoperatively determined 3D bone surface with a coarse point ICP registration and a subsequent model-to-model rigid registration with the SlicerIGT extension in 3DSlicer (Fig. 2iii) [13]. Intraoperative navigation allowed for positional feedback of the tracked surgical pointer in correlation with the preoperative 3D model (Fig. 2v). Moreover, localization can be performed with the iUS superimposed with other available imaging modalities (Fig. 2vi).

### User experience

Intraoperative evaluation was performed by six oncologic pediatric surgeons (CvdV 24 years senior consultant, AvdS 15 years senior consultant, GB three years fellow, STvS 12 years senior consultant, MW 25 years senior consultant and CH eight years senior consultant) and two pediatric oncologic orthopedists (LvdH first year fellow and MvdS 12 years senior consultant). The surgeons were asked to localize one surgical target without the proposed IGS system and ten targets with the help of the IGS system, each operating on a different cadaver. After determining the point of entry, they made an incision and localized the surgical digital target on the bone. Finally, to determine the ease of use and experience of the system, the participating surgeons were asked to score several statements on a five-point Likert scale ranging from ‘totally disagree’ (1/5) up to ‘totally agree’ (5/5).

### Accuracy

The accuracies of the US probe, the tracked surgical pointer and the gold standard were defined as root-mean-square (RMS) errors. During the experiment, a target registration error (TRE) was computed which defines the Euclidean distance between each surgical target located by the gold standard and the iUS registration. Registration was classified as successful if the TRE was lower than 5 mm, as determined by our surgeons based on clinical experience and the surgical margin of > 1 cm.

## Results

### Automatic segmentation

The network predictions on the independent dataset resulted in a median DSC of 0.699 (min 0.57–max 0.80) for all different bone structures. The median coverage percentage was 96% (range 92–98%) and a median distance error of 1.57 mm (range 1.39–1.83 mm). After selection of the largest component and visual inspection, there were no significant protrusions of the segmentation of the bone surface.

### IGS system

The tracked US and tracked surgical pointer were calibrated with a RMS error of 1.00 mm and 0.17 mm, respectively. The gold-standard registration had a mean RMS error of 1.70 ± 0.17 mm. The overall system had a mean TRE of 6.78 ± 1.33 mm. Successful iUS registration (TRE < 5 mm) was found in 3/8 cadavers with a mean TRE of 2.81 ± 1.04 mm. Tracking interference between the iUS probe and the cadaver ORBs was observed in 5/8 cadavers and may result in relatively large TREs. The surgical procedure was interrupted for the iUS sweep only and had a mean time of 2.25 ± 0.70 min. The registration was performed while the surgeon continued with the start of the experiment and the mean computation time of the automatic bone segmentation and the complete registration was 3.70 ± 1.11 min and 15.16 ± 6.78 min, respectively.

### User experience

The surgeons agreed about their willingness for clinical implementation in current clinical practice (Table 1). They mentioned the additional clinical value of iUS in combination with the 3D model for the localization of soft tissue components. Localization of the surgical targets was fast and easy. Moreover, the surgeons were even more confident about the localization with the addition of the IGS system. Finally, they agreed about the advantage of the minimal workflow interruption of the image acquisition, i.e., iUS sweep, which allows for fast intraoperative re-registration in the surgical wound bed.

**Table 1:** Results of the post-procedural questionnaire

Statement		Median	IQR
1	I was confident about my localization of the surgical target	4	2
2	Localization with the aid of IGS was fast	5	1
3	Localization with the aid of IGS was easy	5	0
4	The proposed IGS is worth the additional time for registration	5	1
5	I would like to use the proposed IGS in my current practice	5	0

1 = totally disagree, 2 = disagree, 3 = neutral, 4 = agree and 5 = totally agree

## Discussion

In this study, an iUS-based registration system was developed and evaluated in eight porcine cadavers. The involved clinical panel was satisfied with the relatively fast manner of image acquisition (i.e., less than three minutes), which is in line with the findings of Hiep et al. [8]. Considering the time required for the image acquisition, the surgeons will only be interrupted shortly and can directly continue without navigation till the registration has been completed in the background. Compared to a CT-based registration, the iUS-based acquisition is six-times faster, more accessible (i.e., a percutaneous iUS sweep instead of performing an intraoperative CT), less distracting and does not require additional harmful exposure to radiation for the pediatric patients. Resultingly, the introduction of iUS registration allows the surgeon to perform a relatively simple re-registration during the surgical procedure.

The TRE reported in this study is relatively high compared to the literature [8–12]. Although the clinical desirable accuracy of 5 mm was achieved in three cases, the accuracy of the proposed system must be improved for clinical implementation. However, the possibility of localizing of soft tissue components with iUS-based IGS was emphasized by the surgeons even though the relatively large TRE reported in five cadavers. Improved localization of soft tissue components during OS surgery allows for more surgical confidence during complex surgeries and might improve radical resections, resulting in low local recurrence rates (i.e., sixfold decrease of the local recurrence rate) and thereby improving patient survival [5]. Moreover, the participated surgeons mentioned that the proposed system could also be helpful during the surgical treatment of Ewing sarcoma or chondrosarcoma.

This study has some limitations that might cause the relatively large TRE. First, this study did not involve a speed of sound correction for the US registration in the ex vivo porcine cadaver experimental situation in comparison with the in vivo human situation of the US dataset. Resultingly, a mismatch between the bone surface based on the CT and delineated iUS image of ~ 1–2 mm was detected. Secondly, tracking interference was observed, as the optical tracker had problems to distinguish between the three ORBs. These should be positioned at a greater distance in future setups.

Although the clinical panel underlined the clinical potential and the practical image acquisition, the technical feasibility requires further substantiation. The TRE and registration time could be improved with network improvements, a more heterogenous training datasets and 3D segmentation data. Furthermore, more stable tracking, automatic registration, improved computational hardware and a speed of sound correction might improve the TRE and registration time. These adaptions of the system are considered as future work and as prerequisites for the clinical implementation of this proposed IGS system in the surgical treatment of pediatric OS.

## Conclusion

This study evaluated the potential of an iUS-based registration system using a porcine cadaver model. Surgeons were unanimously enthusiastic about the iUS bone-based registration method and recognized the intraoperative potential for tumor and soft tissue localization in pediatric OS patients. However, several limitations need to be overcome before the proposed system can be clinically implemented.

### Supplementary Information

Below is the link to the electronic supplementary material.

Supplementary file1 (MP4 66220 KB)
